# The anatomical SP-CL stem demonstrates a non-progressing migration pattern in the first year: a low dose CT-based migration study in 20 patients

**DOI:** 10.1080/17453674.2020.1832294

**Published:** 2020-10-16

**Authors:** Olof Sandberg, Simon Tholén, Sofia Carlsson, Per Wretenberg

**Affiliations:** a Sectra AB, Linköping;; b Department of Radiology, Lindesberg Hospital, Örebro University Hospital;; c Department of Medical Sciences, Section of Orthopaedics, Örebro University Hospital, Sweden

## Abstract

Background and purpose — RSA is the gold standard for evaluation of early implant migration. We report the results of a new CT-based method Sectra CT micromotion analysis (CTMA) applied to assess the migration pattern in 20 patients in the 1st year after surgery, both with and without the use of tantalum beads in the bone. The patients had an SP-CL anatomical stem that uses an S-shape, designed to better fit the curvature of the femur.

Patients and methods — 20 THA patients (mean age 61 years, 10 female) received SP-CL stems, tantalum markers in the femur, and low-dose CT scans at 1 day, 3 months and 12 months postoperatively. In addition, precision as well as inter- and intra-observer variability of the 12-month migration was measured.

Results — The 3-month subsidence was median 0.5 mm (95% CI 0.3–1.0) and the internal rotation 1.8° (CI 0.9–2.6). At 12 months the corresponding values were 0.6 (CI 0.3–1.6) mm and 1.9° (CI 0.8–2.4). Precision was 0.1 to 0.3 mm and 0.1° to 0.4° at 3 and 12 months. Intra- and inter- observer variability yielded R-values averaging 0.96 and 0.98.

Interpretation — The migration mainly took place during the 1st 3 months, in line with other uncemented stems. The number of patients with subsidence over 2 mm in the first year (5) might be due to the design of the prosthesis with an anatomical shape. Alternatively, our results might indicate a challenge when choosing the correct size for these new anatomical stems. CTMA provided precise and highly repeatable measurements of migration without the need for tantalum markers.

Anatomical stems use various features such as an S-shape, surface roughness, and grooves to attempt to increase stability and minimize stress shielding and aseptic loosening. Anatomical stems are designed to fill the proximal femur to allow for better osteointegration. This concept has been demonstrated to give good long-term results (Kim 2008). Compared with other anatomical stems, the uncemented anatomical SP-CL stem from Link (Waldemar Link, Hamburg, Germany) has a longer distal part to help the surgeon place the stem in the correct position. Computer simulations have indicated that this stem design should provide considerable advantages for strain shielding (Heyland et al. 2019), but to our knowledge the stem performance has not been evaluated in a migration study.

The long-term survival of an implant can be assessed in a small cohort by using radiostereometric analysis (RSA) (Kärrholm 2012). Problematic implants will already distinguish themselves after 1–2 years through their migration pattern (Pijls et al. 2012). Underperforming designs can be identified and avoided early on (Nelissen et al. 2011).

For the last 3 decades implant migration measurements have primarily been done with RSA. Since 2018 a new CT-based alternative, Sectra CT based Micromotion Analysis (CTMA), has become available. This method relies on standard CT machines instead of the specialized RSA-lab equipment.

We describe the 1-year migration pattern of the SP-CL anatomical stem and assess the capabilities of the CTMA tool.

## Patients and methods

### Patients and surgical procedure

20 THA patients (mean age: 61 years (range 45–74), 10 female) underwent between May 2017 and May 2018 total hip replacement with anatomical stems (SP-CL, Waldemar Link, Hamburg, Germany) and tantalum markers. Inclusion criteria were age 30–85 years and normal anatomy. All patients were ASA class 1 or 2. 2 experienced surgeons performed the operations. Preoperative templating was done in 2D using the Sectra Orthopedic package (Sectra, Linköping, Sweden) to preliminarily plan implant size and positioning with the final decision taken during surgery. All surgeries were done with a posterior approach.

### Implant

The core of the SP-CL stem is made of a titanium alloy. The stem has a polished finish on the distal and proximal parts, with a calcium phosphate coating on the central part, which has a ribbed structure. The implant has an S-shape.

### CT scans

The patients received CT scans postoperatively on day 1, at month 3, and at month 12. The settings used for the CT (Siemens Somatom Definition AS 64 slices) were a kVp of 120 kV, a tube current of 30 Eff mAs, 0.6 mm increments, 1.0 pitch, and a rotation time of 0.5 seconds. Slice acquisition was done in 64 x 0.6 mm. Reconstruction was done with a model-based Safire level 3 I41f algorithm in 0.6 mm thick slices, using IMAR at level Hip Implants. The dosage received per scan was 1.7 mSv. This protocol was based on a previous study (Eriksson et al. 2019).

### Migration measurement

**Figure 1.**
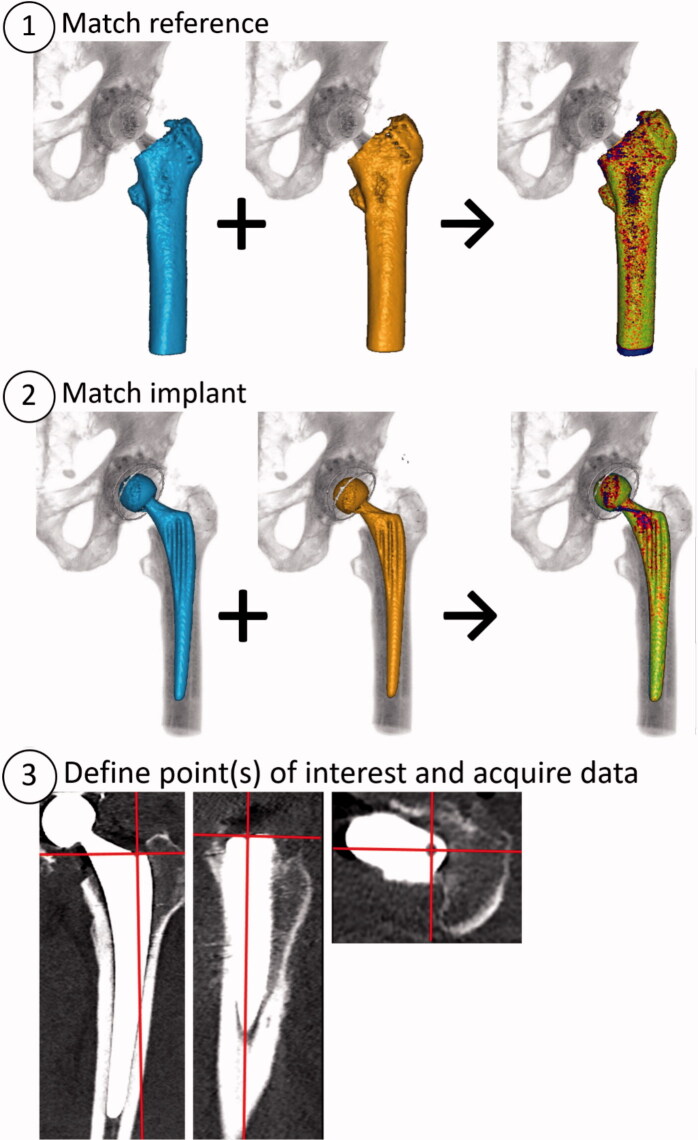
CTMA basic workflow. Step 1: identify the reference body (femur) in both postoperative CT and follow-up CT. The resulting computer-generated blue/red/green color scale indicates how well the computer matched postoperative and follow-up. Step 2 repeats the process but now for the moving body (stem). In the 3rd and final step one or several points of interest are defined. These are the points for which data is to be reported. Definition of a custom coordinate system can also be done in the same step. Data acquisition includes migration quantification as well as moving images.

**Figure 2.**
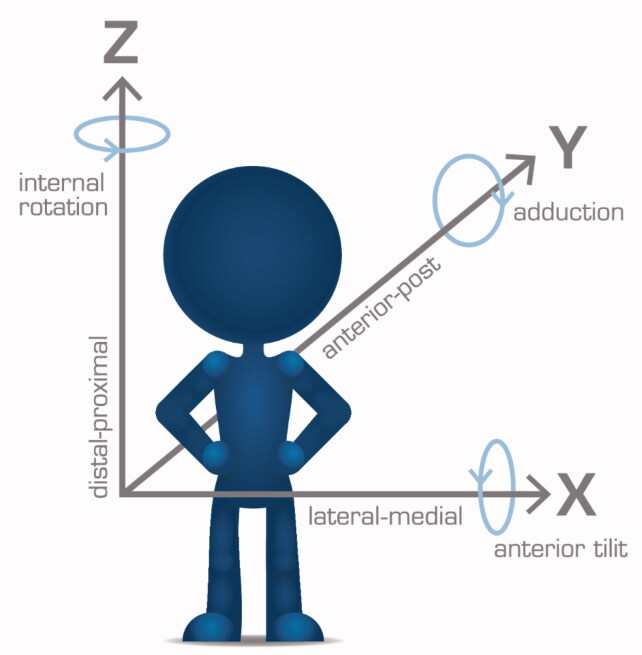
Standard DICOM frame of reference used in this article. Arrows point in the positive direction.

CT scans were loaded into a migration measurement tool (CTMA version 21.1.1, Sectra, Linköping, Sweden). The main procedure followed the steps below (Figures 1 and 2):

1. 2 CTs of the same patient are loaded into the CTMA software (for example postoperatively and 1 year).
2. A reference body (such as the femur) is indicated by the user in both CTs and the software then matches the positions of the body as closely to each other as possible (Figure 1, step 1). A color-coded overlay indicates wellness of fit. The user decides based on visual inspection whether the matching is sufficient, whether it needs correction, or in extreme cases whether it merits exclusion.
3. Step 2 is repeated with a moving body (the stem, Figure 1, step 2).
4. The coordinate system is adjusted (in this study we used standard DICOM, i.e., patient-oriented coordinate system, Figure 1, step 3 and Figure 2).
5. Measurements points to be reported are defined (Figure 1, step 3. This study used tip, neck, and center of the head, see Figure 4, Supplementary data).
6. The software reports the movement and rotational changes for the points of interest relative to the reference body, reported in the chosen frame of reference. The data is imported into a suitable statistics software. Moving images can also be collected (see Supplementary Video).

### Reported quantities and repeatability measurements

The main reported results in this study are internal rotation and subsidence, as they are closely connected to the most common modes of failure. Additional measurements for all 3 migration and rotation directions were made on head, neck, and tip both with and without tantalum markers for the reference body (Figure 5, Tables 4 and 5, Supplementary data). All measurements were done by 2 observers in parallel according to a protocol decided on in advance. One observer (ST) was new to the tool, and the other (OS) was experienced. OS repeated the measurements again after 1 month. The repeated measurements formed the basis of repeatability estimations.

### Exclusion criteria for images of poor quality

If a CTstack deviated from the recommended reconstruction settings, observers 1 and 2 together decided whether an adjustment of Hounsfield unit settings in CTMA could make the CT readable or whether it should be excluded. Default HU settings were 300 for bone and 2,200 for metal.

### Exclusion criteria for marker-based measurements with poor marker spread

In RSA studies a condition number above 150 is typically used as an exclusion criterion for tantalum markers that are insufficiently spread out. Since the CTMA software does not provide this calculation, an RSA expert with over 20 years of experience with RSA was shown the CT stacks of each patient and asked to categorize which patterns could clearly be estimated as “below 150 in condition number.” This was done based on a visual inspection of the CT stacks in 3D visualization software. All patterns that did not fall into this category were excluded from the bead-based data analysis.

### Precision estimates

9 patients had 2 CT examinations taken on the same occasion, so-called double examinations. The precision definition used was based on the ISO standard for RSA with the standard deviation of the double examinations multiplied by a constant to provide the 95% confidence interval around zero (Standardization IOf: International Standard ISO 16087:2013(E)). In our study precision is reported with the formula


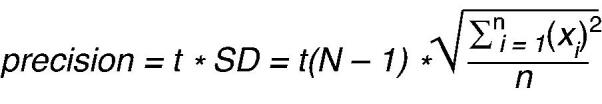


where *t(N-1)* is the value from the t-distribution that corresponds to a 95% double-sided interval with N-1 degrees of freedom where N equals the number of double exams. A t-distribution is considered to give a better estimate than a normal distribution due to N being lower than 30. The chosen expression for SD takes height for the measured quantity of total translation, which is not centered around 0. Mean difference between the double examinations is also reported.

### Statistics

Normality was tested for by looking at Q-Q plots and through a Shapiro–Wilks test. For data sets that were not normally distributed bootstrapping using 1,000 samples was used to generate 95% confidence intervals (CI) of the median. Both inter- and intra -observer variability was tested with a 2-way random intraclass correlation in absolute agreement, single measures mode (ICC(2,1)) R-value calculation and reported with the lower 95% CI. SPSS Statistics subscription version 1.0 (IBM Corp, Armonk, NY, USA) was used for statistics and Graphpad Prism 8.4.0 (Graphpad Prism Software, San Diego, CA, USA) for graphs.

### Ethics, funding, and potential conflicts of interest

This study was approved by the Swedish Ethical Review Authority in Uppsala under approval ID number 219-00117 and funded by Region Örebro County and by Link Sweden AB (Åkersberga, Sweden), which provided institutional support but with no influence on study design or presentation.

PW, ST, and SC report no potential conflicts of interest. OS is a full-time employee at Sectra, a company commercializing a CT-based migration measurement tool.

## Results

No patients or CT exams were excluded from the study. All patients were followed up. There were no complications during surgery and no patients experienced complications. At the time of writing approximately 2 years have passed since surgery and so far no problems have occurred in any patients.

**Figure 3.**
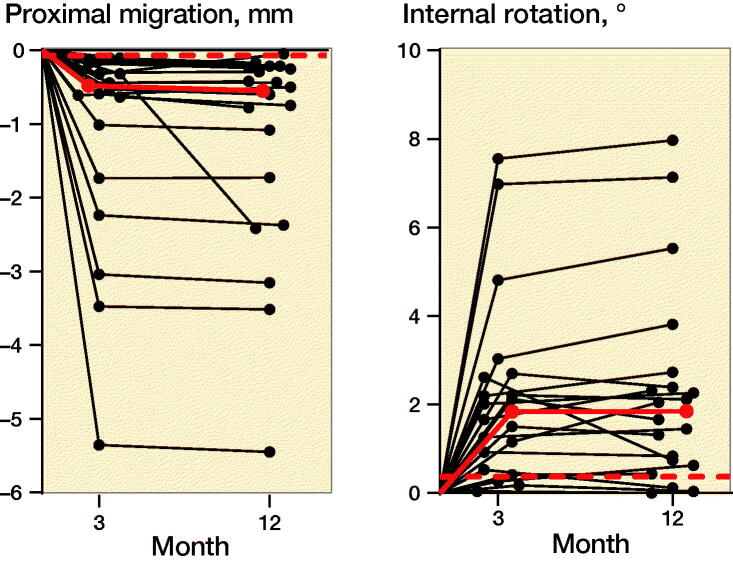
Individual patient values for subsidence and internal rotation of the neck at 3 and 12 months. The median values are shown in red and the precision limit is shown with a dashed red line.

The migration data at 3 and 12 months was mostly found not to be normally distributed and we decided that all migration quantities were treated as such. The median 3- and 12-month subsidence for the neck was 0.5 and 0.6 mm and the internal rotation 1.8 and 1.9° (Table 1 and Figure 3, as well as Video link in Supplementary data). Visual inspection of the data (graph not shown) demonstrated no clear correlation between subsidence and internal rotation.

**Table 1.** Median subsidence and internal rotation of the neck at 3 and 12 months, with 95% CI in parentheses

Factor	3 months	12 months
Subsidence, mm	0.5 (0.3–1.0)	0.6 (0.3–1.6)
Internal rotation, °	1.8 (0.9–2.6)	1.9 (0.8–2.4)

Inter- and intra-observer variability was reported to be 0.88–0.99 and 0.90–0.99 respectively for the different measured quantities and with bone-based or bead-based measurement (Tables 2 and 3).

**Table 3.** Intra-observer variability, reporting the lower 95% CI for the R-value

Location	medial	Translation				Rotation		
		x, posterior	y, proximal	z, translation	TT, total tilt	x, anterior tion	y, adduc- rotation	z, internal
Head								
Bead	0.95	0.95	0.99	0.98	0.90	0.97	0.98	
Bone	0.95	0.99	0.99	0.99	0.98	0.94	0.97	
Neck								
Bead	0.95	0.97	0.99	0.99	0.90	0.97	0.98	
Bone	0.97	0.92	0.99	0.99	0.98	0.94	0.97	
Tip								
Bead	0.95	0.90	0.98	0.90	0.90	0.97	0.98	
Bone	0.96	0.93	0.99	0.99	0.98	0.94	0.97	

**Table 2.** Inter-observer variability, reporting the lower 95% CI for the R-value

Location	medial	Translation				Rotation		
		x, posterior	y, proximal	z, translation	TT, total tilt	x, anterior tion	y, adduc- rotation	z, internal
Head								
Bead	0.99	0.96	0.99	0.98	0.96	0.99	0.98	
Bone	0.97	0.99	0.99	0.99	0.99	0.98	0.98	
Neck								
Bead	0.99	0.95	0.99	0.99	0.96	0.99	0.98	
Bone	0.96	0.95	0.99	0.99	0.99	0.98	0.98	
Tip								
Bead	0.99	0.94	0.99	0.98	0.96	0.99	0.98	
Bone	0.88	0.95	0.99	0.99	0.99	0.98	0.98	

5 of the 69 CT scans have been saved with reconstruction settings differing from the study protocol (deviations in slice thickness, metal artifact reduction, and/or different soft tissue algorithms) but for which adjustments of the parameters for HU extraction were successful in rendering them possible to analyze in all cases. 7 out of 20 patients had bead patterns that could not be assessed as being most likely below 150 in condition number. Therefore these 7 patients had their marker-based measurements excluded from any further analysis.

The precision for subsidence and internal rotation was 0.07 mm and 0.37°. Using the femoral bone rather than the tantalum markers increased precision, especially for rotations. While the head and neck had similar precision, the precision of the tip measurements was worse, in particular when using tantalum markers (Table 5, Supplementary data).

## Discussion

This study describes the migration behavior of the SP-CL cementless anatomical stem, as well as the applicability of the CT-based micromotion measurement tool CTMA. Our results show a median 1-year subsidence of 0.55 mm and that this stem exhibits non-progressive behavior, i.e., that a major part of the migration occur early, prior to the 3rd postoperative month. Thereafter very little additional migration is seen. Similar behavior has been shown in numerous RSA migration studies on other uncemented stems, with follow-up times of up to 10 years (Callary et al. 2012, Weber et al. 2014, Aro et al. 2018, Sesselmann et al. 2018).

Different implant types are thought to have different thresholds as to what is an acceptable level of migration. Another anatomical uncemented stem, ABG II, had a 2-year median subsidence of 0.7 mm (Aro et al. 2018), while other designs of uncemented stems have reported 1-year means of 0.6 mm (Callary et al. 2012), 0.3 mm (Sesselmann et al. 2018), (Weber et al. 2014), and 1.4 mm (Wolf et al. 2010). While less migration is always preferred, the threshold for maximum migration is unknown for an uncemented stem. Progressive migration past the early phase may present the greater risk, as compared with an abating early migration. The shorter uncemented, anatomical CFP stem, which is also designed for metaphysical anchoring with a porous surface structure, has been demonstrated to have a similar non-progressive subsidence behavior, though with smaller absolute values. Röhrl et al (2006) could see a connection between increased subsidence and too small a stem curvature. It has been shown in cemented stems (which intend to recruit absolute stability from the beginning) that a 2-year subsidence of over 1.2 mm implies a 50% risk of revision (Kärrholm et al. 1994). To our knowledge no corresponding long-term follow-up has yet been conducted for uncemented stems.

It is noteworthy that no patients needed to be excluded from the markerless migration analysis. There is no risk for marker occlusion when using CTs, as there is when using RSA images. And while unsatisfactory image quality is a theoretical cause for exclusion, all 69 CT scans and reconstructions performed in this study were of acceptable quality.

1 patient had a deviating migration pattern with continued migration past the third month. At 2 years post-surgery this patient yet had to return with any symptoms or complaints. In a study on uncemented stems Klein et al. (2019) had 2 revisions, both of which exhibited this type of migration.

We used a new CT-based measurement tool. It demonstrated a markerless precision (0.1–0.3 mm and 0.1–0.4°) which compares excellently with the current gold standard RSA where examples of precision values for uncemented stems are reported at 0.1–0.2 mm and 0.3–2.0° (Wolf et al. 2010, Nysted et al. 2014, Weber et al. 2014).

We also included precision measurement of 9 cemented cups as these were visible in the CT stacks. That precision, 0.1–0.2 mm and 0.1–0.2°, is similar to the precision reported in another recently published article on the precision of CTMA for cups: 0.1–0.3 mm and 0.2–0.4° (Brodén et al. 2020b).

Both the stem and the femur have an elongated shape, which possibly might explain the worse precision of the rotation measurement along that axis and hence why the precision values of the stem rotations differ more for the different rotational directions while the precision values of a cup rotations appear to be more similar regardless of the direction.

We observed that tantalum markers did not seem to offer any benefit to precision. On the contrary, the marker-based precision was markedly inferior for almost all translations and rotations. This indicates that, where possible, tantalum markers in the bone should be avoided. That the precision for the tip was markedly worse with bead-based measurements might be that the tantalum markers are placed preferably in the proximal portion of the femur and so are further away from the tip.

This study for the first time reports inter- and intra-observer reliability of CTMA. The results demonstrated that a relatively inexperienced and an experienced user could produce similar excellent results (R-values). This indicates a robustness in the measurement tool.

3 patients had a subsidence of over 3.0 mm at the 1st follow-up. This may have been caused by under-sizing of the implants. 2 years postoperatively none of the patients in the study, including these 3, have returned with any complications. It might be more challenging to correctly template the stem due to the small incremental steps between sizes. This could have been connected to a learning process but the surgeons in our study had performed 10 operations each with this implant prior to study start. Furthermore, a closer look at the distribution of subsidence across the patients indicated no temporal effect, i.e., patients at the end of the series seemed to have had a similar risk of high subsidence as those at the beginning of the series. It has been suggested that anatomical models are less forgiving in certain steps (Giebaly et al. 2016).

CTMA has a number of advantages when compared with RSA: it is not dependent on any markers that have to be added and remain visible and stationary on/in the bone and/or implant. Furthermore, CTMA requires no 3D models of the implants to be acquired from the manufacturers or created via reverse engineering. Neither does it require access to an exclusive RSA lab and image capture does not involve the use of a calibration cage. In theory the CT-based approach could simplify follow-up as the patient does not have to return to the same hospital (though all but one follow-ups in this study were performed at the same hospital).

All CTs used in this study were low dose, which reduces the radiation to below the thresholds set by the European commission on radiation protection in medical research such as this (European Commission 1998).

Our study has limitations. While we chose 1 year as follow-up time, corresponding RSA studies usually have 2 years. Furthermore, we used a new migration measurement tool based on CT, which is not as validated as RSA. However, the CTMA software has previously been tested with good results (Bakhshayesh et al. 2019, Brodén et al. 2020a, b, Eriksson et al. 2019, Schriever et al. 2020) and the underlying principles of CT-based migration measurement have been studied in over 30 scientific publications over the last 2 decades (Olivecrona et al. 2003, 2004, 2016, Polfliet et al. 2015, Scheerlinck et al. 2015, Boettner et al. 2016a, b, Brodén et al. 2016, Otten et al. 2017).

In conclusion, the SP-CL anatomical stem was shown to have a non-progressive migration pattern in line with other uncemented stems, with very little migration past the 3-month mark. The CT-based method was demonstrated to have excellent repeatability and precision. Tantalum markers in the bone were not needed.

## Supplementary Material

Supplemental Material

Supplemental Material

## Supplementary data

A supplementary video is available at http://actaorthop.org/include/sup/14110-video.mp4.

Figures 4–5 and Tables 4–5 are available as supplementary data in the online version of this article, http://dx.doi.org/10. 1080/17453674.2020.1832294
