# Supplementary material for: The anatomical SP-CL stem demonstrates a non-progressing migration pattern in the first year: a low dose CT-based migration study in 20 patients
Source: Acta Orthop. 2020 Oct 16;91(6):654–9. doi: 10.1080/17453674.2020.1832294 (PMC8023890; doi:10.1080/17453674.2020.1832294)
Supplement: Supplemental Material [file IORT_A_1832294_SM5345.pdf]

Supplementary data

Link to Video showing the subsidence and internal rotation of the patient closest to the subsidence median at 12 month (http://actaorthop.org/include/sup/14110-video.mp4)

Table 4. Translation and rotation of the SP–CL stem, median and 95% CI at 3 and 12 months for head, neck, and tip: bone registration in 20 cases without any tantalum markers used

Location Month	Translation, median (95% CI), mm x, medial	y, posterior	z, proximal	TT, total translation	Rotation, median (95% CI), degrees x, anterior tilt	y, adduction	z, internal rotation
Head							
3	0.25 (0.05–0.41)	0.98 (0.59–1.93)	–0.87 (–1.50 to –0.54)	1.98 (1.08–2.29)	–0.04 (–0.29 to 0.01)	0.27 (0.01–0.54)	1.84 (0.93–2.62)
12	0.23 (0.05–0.45)	1.14 (0.59–2.21)	–0.90 (–1.68 to –0.45)	2.08 (1.00–2.55)	–0.08 (–0.17 to –0.01)	0.21 (–0.05–0.30)	1.85 (0.75–2.39)
Neck							
3	0.05 (–0.06–0.23)	0.09 (–0.08–0.21)	–0.48 (–1.01 to –0.31)	0.70 (0.41–1.39)	–0.04 (–0.29 to 0.02)	0.27 (0.01–0.54)	1.84 (0.93–2.62)
12	0.11 (–0.07–0.27)	0.07 (–0.08–0.15)	–0.55 (–1.58 to –0.25)	0.80 (0.43–1.67)	–0.08 (–0.17 to –0.01)	0.21 (–0.05–0.30)	1.85 (0.75–2.39)
Tip							
3	–0.05 (–0.19–0.19)	0.06 (–0.27–0.22)	–0.57 (–1.02 to –0.27)	0.84 (0.50–1.52)	–0.04 (–0.29 to 0.01)	0.27 (0.01–0.54)	1.84 (0.93–2.62)
12	0.05 (–0.11–0.27)	0.03 (–0.13–0.19)	–0.68 (–1.67 to –0.26)	0.87 (0.41–1.76)	–0.08 (–0.17 to –0.01)	0.21 (–0.05–0.30)	1.85 (0.75–2.39)

Table 5. Precision data for measurements of different points (head, neck, tip) on the stem with either bone, or beads in bone, used as the frame of reference. Also includes the precision of the cup versus pelvic bone. Presenting upper 95% CI precision data as well as mean

				Translation, mm								Rotation, degrees					
				x, medial		y, posterior		z, proximal		TT, total translation		x, anterior tilt		y, adduction		z, internal rotation	
Location		n	Reference	CI	Mean	CI	Mean	CI	Mean	CI	Mean	CI	Mean	CI	Mean	CI	Mean
Stem	Head	6	Bead	0.45	–0.09	0.76	–0.03	0.32	0.02	0.94	0.31	0.50	0.01	0.44	0.02	0.68	–0.16
		9	Bone	0.09	–0.01	0.24	–0.01	0.06	–0.01	0.27	0.11	0.08	–0.01	0.06	0.01	0.36	–0.02
	Neck	6	Bead	0.31	–0.06	0.25	0.03	0.06	–0.01	0.40	0.14	0.50	0.01	0.44	–0.05	0.68	–0.07
		9	Bone	0.06	–0.01	0.07	0.01	0.07	–0.01	0.12	0.05	0.08	–0.01	0.06	–0.01	0.36	–0.03
	Tip	6	Bead	0.87	0.01	1.12	0.07	0.19	–0.03	1.43	0.44	0.50	0.01	0.44	–0.05	0.68	–0.07
		9	Bone	0.19	–0.01	0.17	0.02	0.07	–0.01	0.26	0.09	0.08	–0.01	0.06	–0.01	0.36	–0.03
Cup	COM	9	Bone	0.07	0.01	0.17	0.05	0.14	–0.02	0.23	0.07	0.19	–0.09	0.21	0.04	0.07	0.02

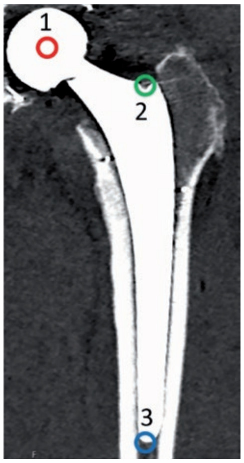

Figure 4. CT slice showing the 3 measurement points: center of head (1), neck (2), and tip (3).

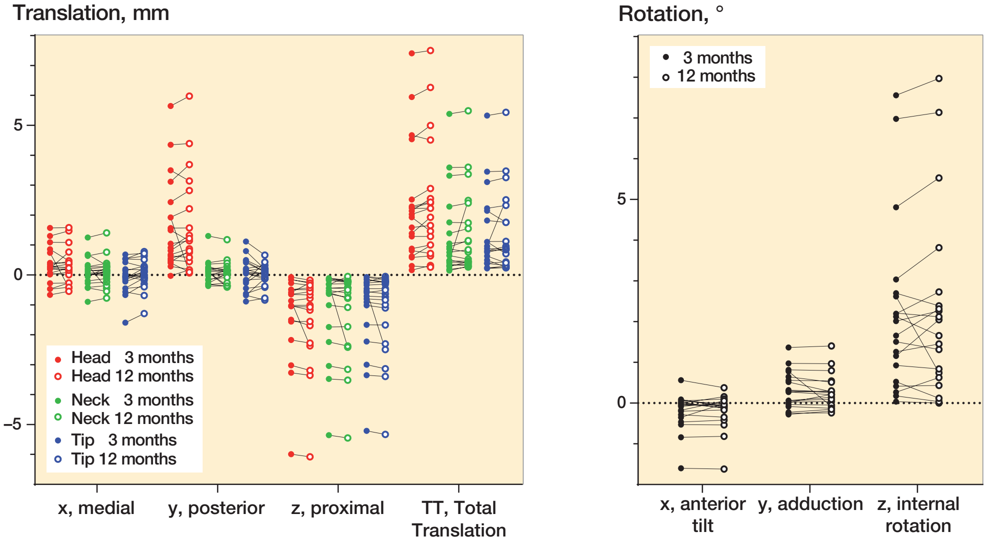

Figure 5. Translation and rotation for neck, head, and tip for individual patients at 3 and 12 months.
